# Tracking Blood-Forming Stem Cells through Development

**DOI:** 10.1371/journal.pbio.0020083

**Published:** 2004-03-16

Of the 200-plus different types of cells that form the mammalian body, most have a finite life span. Like nearly everything in biology, there are exceptions—neurons and muscle cells, for example, can last a lifetime—but the vast majority of cells eventually wear out and must be replaced. Among the most short-lived cells, blood cells are generated continuously, mainly in the bone marrow of an adult, recharging the bloodstream as their depleted predecessors are efficiently dispatched and removed from circulation every 120 days. Some 2.5 million new red blood cells are generated every second from a small pool of stem cells.[Fig pbio-0020083-g001]

**Figure pbio-0020083-g001:**
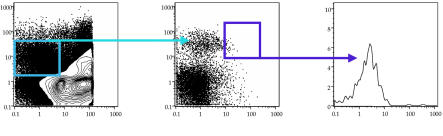
Fluorescence activated cell sorting is used to study the early development of hematopoietic stem cells

Blood cell development, called hematopoiesis, passes through discrete stages in specific tissues in the developing embryo before converging in the bone marrow, where it continues throughout adulthood. Some researchers have proposed that hematopoietic stem cells (HSC) flood the bloodstream during short, precise intervals to build the developing hematopoietic system (which includes the liver, bone marrow, spleen, tonsils, and lymph nodes). Presenting an alternative model for HSC migration, Julie Christensen and her colleagues in Irving Weissman's lab at Stanford University report that HSC in mice gradually leave the fetal liver to colonize the developing spleen and bone marrow as the organs acquire the means to support them.

In mouse embryos, HSC precursors develop first in the yolk sac and a region called the aorta-gonad-mesonephros (AGM), then they migrate to the liver, and later to the spleen, before finally settling into the bone marrow just before birth. It was thought that this migration occurs in distinct waves of HSC production because HSC numbers decrease in one region just before increasing in newly forming hematopoietic sites. Analyzing the concentration and activity of HSC, Christensen et al. found the cells in the blood at low but fairly constant levels during much of late fetal development, when they migrate from the liver to the spleen and bone marrow. Although the HSC population decreases in the liver at 15.5 days after conception, the authors propose that this drop occurs primarily because the HSC have differentiated into mature blood cells, not because they've exited the liver en masse to help build the spleen and bone marrow. On the other hand, the slight decrease in circulating HSC, which also occurs around this time, may be attributed to their recruitment from the bloodstream to these developing tissues.

Christensen et al. also examined the impact of intercellular signaling proteins called chemokines, which help regulate fundamental developmental processes, on HSC migration. To effectively “seed” developing tissues, HSC must first be recruited from the blood, guided to the appropriate nascent tissue, then corralled and sustained. The chemokine SDF-1 attracts and retains HSC in the bone marrow but was thought to have a lesser effect on fetal liver HSC. Christensen et al. demonstrate not only that liver HSC migrate in response to this chemokine, but that their migratory response increases dramatically when both SDF-1 and a signaling protein called steel factor (SLF) are present. While adult marrow HSC respond to SDF-1, they do not respond to SLF alone and do not show improved migration in the presence of both SLF and SDF-1.

Bone marrow transplants have become increasingly common for a number of hematological disorders, including leukemia and aplastic anemia. Since hematopoiesis occurs primarily in the bone marrow in both mice and humans after birth, these findings offer valuable insights into the migratory behavior of these stem cells and suggest how HSC migration might be applied to bone marrow transplants and other clinical therapies.

